# Low Power Resistive Oxygen Sensor Based on Sonochemical SrTi_0.6_Fe_0.4_O_2.8_ (STFO40)

**DOI:** 10.3390/s150717495

**Published:** 2015-07-20

**Authors:** Alisa Stratulat, Bogdan-Catalin Serban, Andrea de Luca, Viorel Avramescu, Cornel Cobianu, Mihai Brezeanu, Octavian Buiu, Lucian Diamandescu, Marcel Feder, Syed Zeeshan Ali, Florin Udrea

**Affiliations:** 1 Honeywell Romania SRL, Sensors and Wireless Laboratory Bucharest (SWLB), Bucharest 020339, Romania; E-Mails: alisa.stratulat@honeywell.com (A.S.); viorel.avramescu@honeywell.com (V.A.); cornel.cobianu@honeywell.com (C.C.); mihai.brezeanu@honeywell.com (M.B.); octavian.buiu@honeywell.com (O.B.); 2 Centre for Advanced Photonics and Electronics (CAPE), University of Cambridge, Cambridge CB3 0FA, UK; E-Mails: ad597@cam.ac.uk (A.L.); fu@eng.cam.ac.uk (F.U.); 3 National Institute of Materials Physics, Bucharest-Magurele, P.O. Box. MG-7, Magurele 77125, Romania; E-Mails: diamand@infim.ro (L.D.); mfeder@infim.ro (M.F.); 4 Cambridge CMOS Sensors Ltd., Cambridge CB4 0DL, UK; E-Mail: zeeshan.ali@ccmoss.com

**Keywords:** sonochemistry, STFO, oxygen sensing, silicon-on-Insulator, CMOS-compatible, dip-pen nanolithography, harsh environment

## Abstract

The current paper reports on a sonochemical synthesis method for manufacturing nanostructured (typical grain size of 50 nm) SrTi_0.6_Fe_0.4_O_2.8_ (Sono-STFO40) powder. This powder is characterized using X ray-diffraction (XRD), Mössbauer spectroscopy and Scanning Electron Microscopy (SEM), and results are compared with commercially available SrTi_0.4_Fe_0.6_O_2.8_ (STFO60) powder. In order to manufacture resistive oxygen sensors, both Sono-STFO40 and STFO60 are deposited, by dip-pen nanolithography (DPN) method, on an SOI (Silicon-on-Insulator) micro-hotplate, employing a tungsten heater embedded within a dielectric membrane. Oxygen detection tests are performed in both dry (RH = 0%) and humid (RH = 60%) nitrogen atmosphere, varying oxygen concentrations between 1% and 16% (v/v), at a constant heater temperature of 650 °C. The oxygen sensor, based on the Sono-STFO40 sensing layer, shows good sensitivity, low power consumption (80 mW), and short response time (25 s). These performance are comparable to those exhibited by state-of-the-art O_2_ sensors based on STFO60, thus proving Sono-STFO40 to be a material suitable for oxygen detection in harsh environments.

## 1. Introduction

In harsh environment applications, especially at high relative humidity levels and at high ambient temperature levels [1], metal oxide-based resistive oxygen sensors are an inexpensive alternative to the well-known potentiometric zirconia oxygen detectors. The development of a resistive oxygen sensor has been a long-standing goal. Even though the research on this topic started more than 40 years ago, there is still no material that is commonly accepted as being the best solution. To date, high power consumption is one of the most important drawbacks of resistive sensors.

New approaches on material synthesis, design and manufacturing, meant to improve parameters, such as temperature independence, response time, cross-sensitivity, long-term stability and power consumption, constantly emerge. In the first stage of development, oxygen detectors employing, as sensing layers, semiconducting metal oxides, such as TiO_2_, CeO_2_, SnO_2_, Ga_2_O_3_, and WO_3_, were fabricated. Their sensing mechanism, explained by the Krӧger and Vink model [2], is based on the reaction between the oxygen vacancies in their structure and oxygen gas. These structures are thermally sensitive (their conductivity varies with temperature), which makes them unsuitable for high temperature operation. Metal oxides with ABO_3_ perovskite structure (BaTiO_3_, LaFeO_3_, and SrTiO_3_) were also studied. Although these materials can accommodate large levels of dopants without displaying phase transformations, they also exhibit a strong temperature-dependence of the resistance response.

Recently, doped perovskites have been studied as promising alternatives for use in manufacturing of oxygen sensors. Their conductivity does not vary with temperature for certain temperature ranges [3]. Among doped perovskites, SrTi_1-x_Fe_x_O_3-δ_ (STFOx) received a particular attention [4,5]. Its resistance has zero temperature coefficient in the 450°C–650°C range. Matrix nanocomposites comprising STFOx and different carbon-based nano-structures (single-wall, double-wall, and multi-wall carbon nanotubes, graphene, fullerene-C60, fullerene-C70, nanobuds, carbon nanofibers) were also proposed as sensing layers for oxygen detection [6].

Different methods for obtaining micro/nano-structured STFOx were developed. Electrospinning [7], microwave-assisted hydrothermal method [8], co-precipitation [9], and self-propagating high-temperature synthesis [10,11,12] were employed for obtaining several types of STFOx (x = 0–0.6) nanofibers, nanocubes and (nano)-powders (particles size in the 40 nm–1.5 µm range). All these methods require thermal treatments performed at temperatures above 1000 °C. The hydrothermal method allows the synthesis of STFOx at lower temperatures (below 1000 ^°^C), but the standard synthesis route in this case is highly complex [13].

Sonochemistry [14] is a versatile method for obtaining nanostructured materials with controlled size and morphology [15,16]. It can be used in the synthesis of materials, such as polymers, biomaterials, inorganic materials, nanostructured oxides, carbides, and sulfides [17]. It can also be employed for nanostructure alignment and controlled growth of nanostructured materials on different substrates [15].

The ultrasound energy emitted by a piezoelectric generator to a liquid yields an acoustic cavitation [18] having bubbles characterized, within their small volumes, by high pressure (up to 1000 bar) and high local temperature (up to 5000 K). It is the water bubble collapse during transient cavitation which provides the driving force for free radicals generation like hydrogen atoms (H•) and hydroxyl (•OH), and for enhancing the physicochemical processes taking place within the bubble in its vicinity. The increase of the hydrolysis rate [15] can produce unique morphological changes. By exposing the solution to ultrasonic irradiation, the reaction kinetics is increased, the running time is decreased, and the sintering process is simplified, by eliminating the need for a solid state reaction between powder precursors. A detailed review of sonochemistry, cavitation, and sonochemical literature is given by Thompson *et al.* [19].

Sonochemistry has the advantage of producing high surface area materials with uniform particle size and better phase purity by increasing the reaction time [15]. The fact that nanostructured materials with different morphologies can be easily obtained by changing the reaction conditions makes sonochemistry one of the most powerful synthesis methods.

Sonochemical preparation of nanoporous composites of strontium titanate (STO), titanium dioxide, nanoceria, and ceria-zirconia oxide solid solutions were reported in literature [20]. Herein, a simple sonochemical method [21,22,23] to synthesize nanocrystalline STFOx (x = 0.4) is presented. The oxygen sensing properties of the new synthesized material are evaluated using a resistive sensor with low power consumption.

## 2. Experimental Section

The first step for synthesizing SrTi_0.6_Fe_0.4_O_3_ (Sono-STFO40) powder [23] was mixing high purity Sr(NO_3_)_2_ and Fe(NO_3_)_3_ (both provided by Sigma Aldrich) with 5 ml of deionized water. The obtained solution was further mixed with a 99.5% TiO_2_ nanopowder (21 nm Transmission Electron Microscopy (TEM) primary particle sized, Sigma Aldrich) to obtain a homogeneous solution. Separately, a 4 M concentration of NaOH solution was prepared by dissolving NaOH pellets (p.a., Merck) in deionized water. NaOH solution was slowly dripped into the nitrates solution, under continuous stirring. Solution preparation was performed under a dry box in a controlled RH atmosphere (~11%) to minimize CO_2_ exposure. The obtained mixture (pH ~14) was sonicated for 1.5 h (~94 W/cm^2^ intensity) in Argon (5 L/min flow) using a Hielscher UP200St (200 W, 26 kHz) ultrasonic generator with a titanium 14 mm sonotrode. The temperature inside the reaction beaker was kept below 75 °C by using an ice water bath (approximately 0 °C).

Salt suspensions and impurities in the final solution were dissolved through distilled water washing until a pH value of 6.5 was reached. Then, the powders were filtrated, dried in air at a temperature of 90 °C and annealed at 1000 °C for 2 h.

Phase identification of Sono-STFO40 was performed at room temperature by employing a DRON-2 diffractometer using CuK_α_ (l = 1.54067) radiation and a graphite monochromator in the diffracted beam, Inorganic Crystal Structure Database (ICSD) files together with Rietveld refinements of the obtained diffractograms. A step scanning technique was applied with a step width of 0.02° in the 2θ range of 20–70°. ^57^Fe Mössbauer spectra were recorded at room temperature on a WissEL-ICE Oxford Mössbauer cryomagnetic system. The source was ^57^Co in Rhodium matrix with activity of ~10 mCi. The absorber thickness was ~7 mg Fe cm^−2^. Sono-STFO40 powders were also characterised by scanning electron microscopy (SEM, Raith e-LiNE plus). Results of these investigations are presented in the next section.

In order to produce an O_2_ sensing layer, Sono-STFO40 slurry was required. Sono-STFO40 slurry was obtained by mixing Sono-STFO40 (powder, 60% w/w), terpineol (solvent, 30% w/w), ethyl cellulose (binder, 5% w/w) and capric acid/caprylic acid (equimolecular mixture, surfactant, 5% w/w).

**Figure 1:**
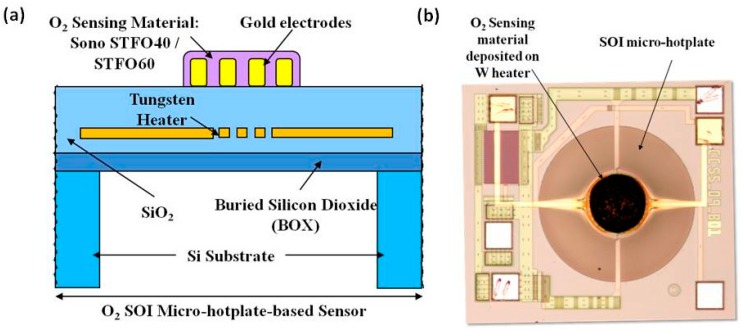
(**a**) O_2_ resistive sensor structure employing a CMOS-compatible SOI micro-hotplate as substrate and Sono-STFO40 as sensing layer; (**b**) Top-view of the manufactured O_2_ resistive sensor.

In order to manufacture the resistive oxygen sensor, the Sono-STFO40 slurry was deposited on a Silicon-on-Insulator (SOI) micro-hotplate membrane, similar to the one depicted in Figure 1. The circular membrane (600 µm diameter) comprises a buried oxide (BOX) layer (1 µm thick), a SiO_2_ layer (~4 µm thick) and a tungsten heater. The silicon substrate was back etched by Deep Reactive Ion Etching (DRIE) to thermally isolate the membrane. The micro-hotplate thus obtained is fully Complementary Metal-Oxide-Semiconductor (CMOS) compatible. Post-CMOS deposited gold interdigitated electrodes (IDEs) allow electrical contact to the semiconductor sensing layer.

The tungsten heater can be safely operated at temperatures up to 650 °C [24]. Other key advantages of the SOI micro-hotplates are their very low power consumption and high temperature uniformity across the heater sensing area [25]. The use of a commercial CMOS process means that the devices can be fabricated in high volume (millions), at low unit costs.

Sono-STFO40 was deposited onto the CMOS microhotplates with a dip pen nano-lithograpy (DPN) system (NLP2000 by NanoInk). DPN is a scanning-probe lithographic technique [26], with possible sub-micron resolution, enabling the deposition of a variety of materials even onto substrates with irregular morphologies (e.g., rounded surfaces), often hindering other deposition methods. DPN is traditionally used for its excellent in-plane resolution in the delivery of picolitres of materials. Herein, we investigate DPN as a fragile CMOS membrane based microhotplates-compatible deposition technique, taking advantage of the high DPN system off-plane resolution and the cantilever-type pens’ mechanical compliance, in order to deposit Sono-STFO40 slurry on a relatively wide area (~200 µm diameter) at once without damaging the membrane. Such a wide deposition area was achieved by using an array of four cantilevers, which were few microns apart from each other. Each cantilever is approximately 50 µm wide and 150 µm long. By entirely dipping the cantilevers in the material reservoir it is possible to load and then deposit enough slurry to coat (by “brushing”) the whole IDEs area.

**Figure 2:**
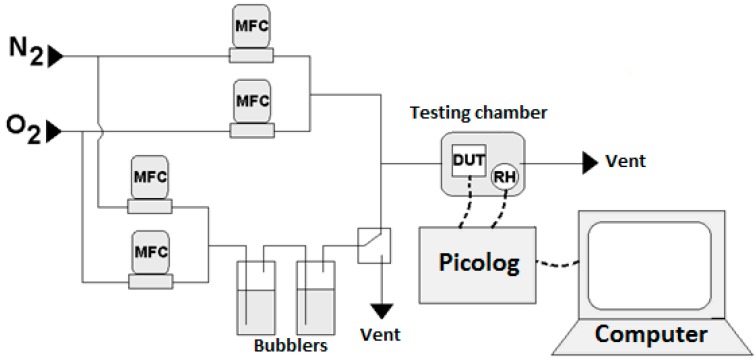
Diagram of the experimental setup employed for O_2_ detection measurements.

Oxygen detection capabilities of the manufactured sensors, employing as sensing layer either Sono-STFO40 or STFO60, were measured by using an in-house experimental setup, shown in Figure 2. The setup comprises a small testing chamber (5 mm × 5 mm × 5 mm) with gas inlet/outlet and electrical connections, a system of mass flow controllers (Brooks MFC 4800 Series), two glass bubblers (for relative humidity (RH) control), a PicoLog and a computer for data readout. The oxygen sensor was encapsulated in a TO-5 package (shown as device under test (DUT) in Figure 2) and placed in the testing chamber together with a RH sensor.

## 3. Results and Discussion

**Figure 3:**
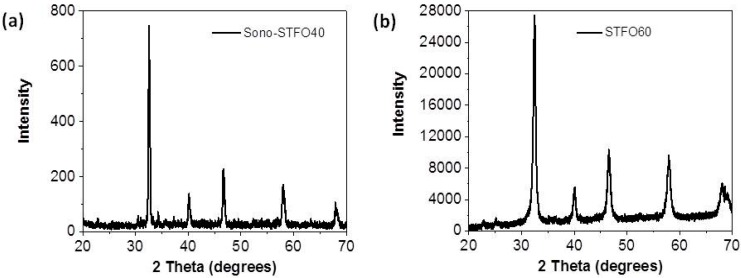
X-ray diffractogram of (**a**) the Sono-STFO40 and (**b**) commercially available STFO60.

For the Sono-STFO40 powder synthesized as described in the previous chapter, the Rietveld refinement of the X-ray diffractogram (XRD) in Figure 3a indicates that the composition is the following: 97.55% Sr(Ti_0.6_Fe_0.4_)O_2.845_ and 2.45% SrFe_12_O_19_. The average crystallite size is approximately 50 nm. For the purpose of comparing the structure and O_2_ detection performance of the Sono-STFO40 with the STFOx state-of-the-art, an XRD analysis (Figure 3b) was also performed on commercially available SrTi_0.4_Fe_0.6_O_2.8_ (STFO60) produced by Neri *et al.* [10,11,12].

**Figure 4:**
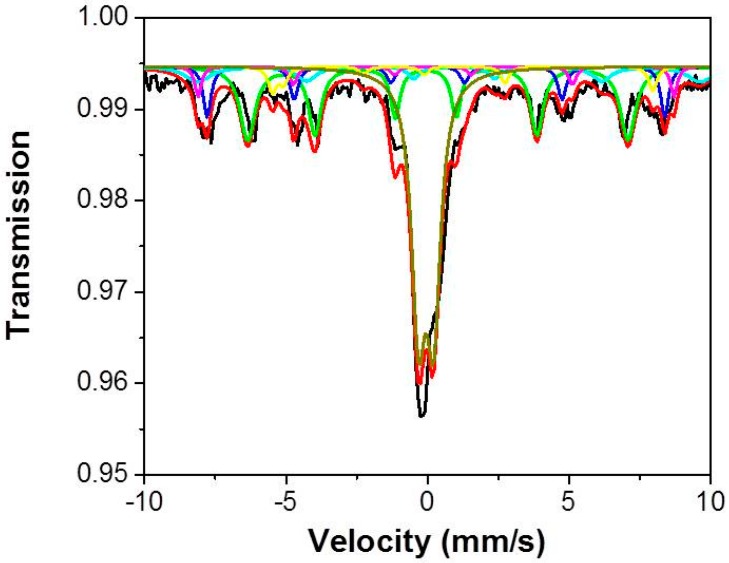
The Mössbauer spectrum of Sono-STFO40.

The room temperature Mössbauer spectrum (Figure 4) of Sono-STFO40 consists in the superposition of two components: a prevailing central quadrupole doublet (continuous line in blue) assigned [27] to Sr(Ti_0.6_Fe_0.4_)O_2.845_ and a complex magnetic hyperfine component associated with five iron sites (12k, 4f_1_, 4f_2_, 2a and 2b) in the SrFe_12_O_19_ structure [28] (continuous color sextet lines). The continuous lines in Figure 4 represent the computer fit of the experimental points in the hypothesis of Lorentzian line shape. This result is in good agreement with the XRD data.

**Figure 5:**
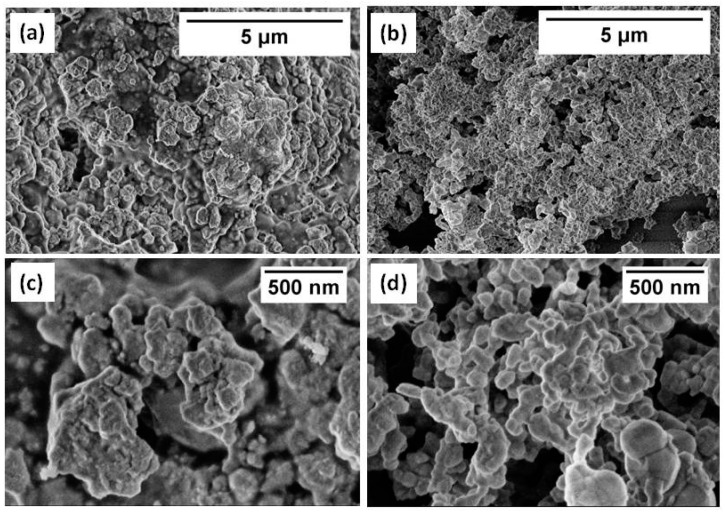
SEM micrographs of (**a**) commercial STFO60 (10 kX); (**b**) Sono-STFO40 (10 kX); (**c**) commercial STFO60 (50 kX); (**d**) Sono-STFO40 (50 kX).

SEM micrographs of both commercially available STFO60 and Sono-STFO40 slurries are shown in Figure 5. The particles are similar in size (nanometric) and have an almost uniform distribution. Nanoparticles agglomerations can be noticed in both types of STFOx. The Sono-STFO40 powder seems slightly more porous than STFO60.

In order to assess the O_2_ sensing properties of both layers, they were deposited on SOI micro-hotplates, as described above. In order to maximize the oxygen detection sensitivity and to reduce the sensor response time, the tungsten heater within the SOI micro-hotplate was set at a high temperature level (~650 °C). Owing to the clever design of the SOI micro-hotplate, this high temperature level corresponds to a power consumption value as low as 80 mW.

**Figure 6:**
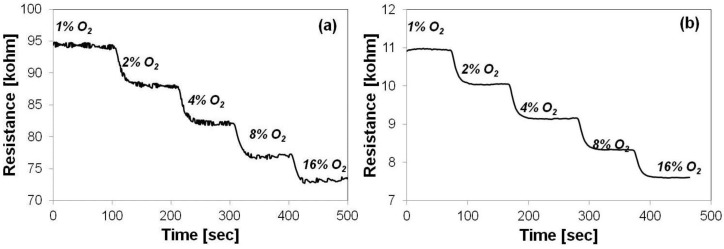
Oxygen sensor response in dry nitrogen atmosphere (1%–16% O_2_) for: (**a**) Sono-STFO40 and (**b**) STFO60.

The sensing capabilities of the two layers are depicted in Figure 6. The Sono-STFO40-based sensor exhibits a resistance one order of magnitude higher than its STFO60 counterpart. This is due to the lower thickness of the Sono-STFO40 layer (~1 µm), owing to the lower viscosity of the slurry. The thickness of the STFO60 layer was measured to be 3 µm. Another reason for the difference in resistivity is related to the Fe doping level, which is larger in STFO60 than in Sono-STFO40, thus inducing a decrease in resistance.

At the same time, the Sono-STFO40 layer exhibits a noisier signal. This is due to its higher resistance value mentioned above. Future work will optimize the Sono-STFO40 synthesizing route in order to obtain thicker layers yielding less-noisier signals, similar to those measured on STFO60-based structures (Figure 6b).

On the other hand, the shape of the response signal exhibited by the Sono-STFO40 layer indicates the possibility that the resistance fluctuations might have a more significant role in oxygen detection than the change in the dc level of the resistance [29]. Further investigation on the role played by the size and density of the nanoparticles and clustered nanoparticles within the Sono-STFO40 layer in its oxygen detection capability will be performed. The influence of the Sono-STFO40 layer thickness on the level of resistance fluctuations will also be assessed.

The response time (t90) of both sensors has been assessed while switching the oxygen concentration from 2% to 4% in dry nitrogen atmosphere. Both tested devices exhibited a response time of less than 25 s.

STFOx behaves as a semiconductor, exhibiting both electron- and hole-based electric conduction. The conductivity, *σ*, is correlated with the oxygen partial pressure in accordance with the:

(1) $$\sigma \;\alpha \;p{\left(O_{2}\right)}^{m}$$

law, where *p(O_2_)* is the oxygen partial pressure and *m* is a coefficient that has a theoretical value of ±0.25. Pending on the conductivity type, *m* coefficient is either negative or positive. For low oxygen partial pressure, the STFOx behaves as an n-type semiconductor having *m = −*0.25. For oxygen partial pressure above ~10^−5^ bar, the STFOx behaves as a p-type semiconductor, and the conductivity increases with the oxygen concentration.

In theory, the value of *m = ±*0.25 has been computed for a monocrystal. However, in reality, any material has a disordered structure. This disorder decreases the material sensitivity towards oxygen. This leads to *|m|* values lower than 0.25.

**Figure 7:**
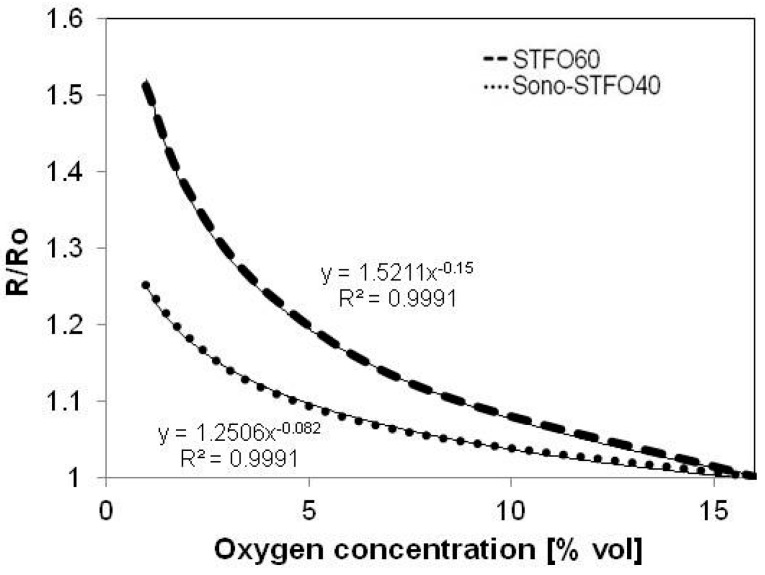
Normalized resistance *vs.* oxygen concentration for both Sono-STFO40 and STFO60.

Figure 7 shows the layer resistance variation with the O_2_ concentration for both STFO60 and Sono-STFO40. The reference resistance (R_0_) is the resistance measured at an oxygen concentration of 16% in dry nitrogen atmosphere. Exhibiting *m =* 0.082, the Sono-STFO40 layer proves to be less “crystalline” than STFO60 (*m =* 0.15) and thus more “disordered”. Both materials show good sensitivity towards oxygen, with a slight advantage for STFO60. These results might be due to the higher temperature employed to synthesize STFO60, leading to a more coherent material microstructure.

Gerblinger *et al.* [30] state that a way to improve the O_2_ sensitivity for SrTiO_3_ (STO) layer, pristine or Fe-doped, is to employ a higher annealing temperature of the as-deposited material. In the case of Gerblinger’s study, the value of *m* corresponding to an STO layer was shown to increase from 0.16 to 0.22 after annealing the sensing material at 1300 °C for 15 h [29]. In the case of our study, more investigations are needed to confirm these assumptions.

**Figure 8:**
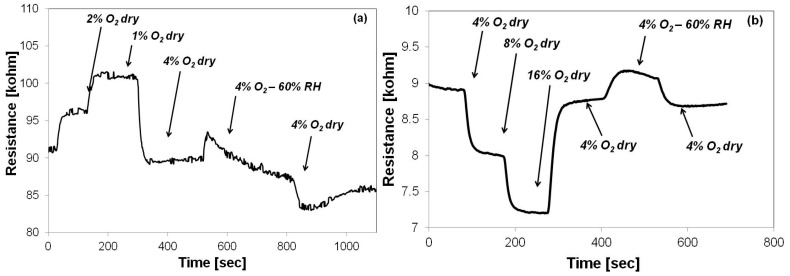
Sensor response in dry (RH = 0%) and humid nitrogen environment (RH = 60%) when employing (**a**) Sono-STFO40 and (**b**) STFO60, as sensing layers.

In order to assess their availability for operation in harsh environments, the oxygen sensing capabilities of the two materials have also been measured compared in a humid environment. Figure 8 presents the sensing layer resistance behavior when varying the RH level from 0% to 60% in a nitrogen atmosphere, while maintaining a constant O_2_ level (4%).

In both cases, when switching from dry to humid and then back, from humid to dry environment, the resistance has a rather small variation: 2% for Sono-STFO40, 4% for STFO60. On the other hand, the resistance of the Sono-STFO40 layer exhibits a more significant drift in time. When turning from the dry to the humid atmosphere, both of the sensing layers lose electric charge carriers, mainly holes, which causes a decrease in conductivity. During the humid plateau region, the conductivity starts to recover: slowly in the case of STFO60, faster for Sono-STFO40. The steep change in conductivity, observed at the end of the humid plateau, might be related to a surface conduction mechanism, due to species produced by the water decomposition occurring in contact with the hot sensing layer. The recovery behavior is probably due to a bulk mechanism that depends on the diffusion of the aforementioned species through the sensing layer until a steady state is obtained. All in all, experimental measurements show that both layers are suitable for operation in environments with relative humidity levels as high as 60%.

## 4. Conclusions

A Sono-STFO40 powder was synthesized using a sonochemical method. When deposited on a CMOS-compatible SOI micro-hotplate employing a tungsten heater, the Sono-STFO40 layer showed oxygen-sensing capabilities comparable to that exhibited by a commercially available STFO60 layer. A low power consumption (80 mW) and fast (25 ms) resistive oxygen sensor, able to reliably operate in environments with relative humidity ranging from 0% to 60%, was thus demonstrated.

The microstructure of the Sono-STFO40 layer is currently subject to improvement work, by varying the conditions of its sonochemical synthesis route. At the same time, methods to perform the annealing of Sono-STFO40 after its deposition on the SOI micro-hotplate at temperatures higher than 650 °C are investigated. The purpose of this work is to reduce the drift of the sensor output signal.

The experimental results reported in this paper, measured a CMOS-compatible oxygen detector, are an important milestone towards obtaining low power sensing structures and systems-in-package addressing highly relevant harsh environment applications, such as combustion optimization and emission monitoring in automotive, aerospace, and domestic and industrial boilers.
